# The informational dysregulation framework of addiction (IDFA): an information-processing model of relapse in opioid use disorder

**DOI:** 10.3389/fpsyt.2026.1819543

**Published:** 2026-07-13

**Authors:** Ovie Martin Albert

**Affiliations:** 1 Department of Family Medicine, Cumming School of Medicine, University of Calgary, Calgary, AB, Canada; 2 Virtual Opioid Dependency Program, Centennial Centre for Mental Health and Brain Injury, Recovery Alberta/Alberta Health Services, Ponoka, AB, Canada

**Keywords:** active inference, computational psychiatry, interoception, neural complexity, opioid use disorder, predictive processing, relapse, relapse prevention

## Abstract

Opioid use disorder is associated with high relapse risk and a persistent mismatch between intention and behavior. Contemporary neurobiological models have clarified the roles of reward learning, reinforcement, habit formation, salience attribution, stress adaptation, and executive-control dysfunction in relapse vulnerability. However, these mechanisms are not always translated into a clinically usable framework that links neurobiology with lived experience and relapse-prevention planning. The Informational Dysregulation Framework of Addiction (IDFA) was developed in response to this translational need, through structured integrative synthesis of addiction neuroscience, computational psychiatry, information theory, and clinical relapse research. IDFA conceptualizes relapse vulnerability in opioid use disorder as dysregulation in how the brain predicts, updates, and integrates information under uncertainty. The framework organizes relapse processes across three interacting domains: precision dysregulation, entropy and complexity disruption, and awareness and integration impairment. Together, these processes form a self-reinforcing loop that narrows informational bandwidth and behavioral flexibility across relapse trajectories. Alongside established reward- and habit-based accounts, IDFA provides an integrative information-processing perspective on how prediction, updating, and action selection become dysregulated during relapse vulnerability. This approach also generates clinically relevant hypotheses regarding relapse prediction, individualized case formulation, and mechanism-informed intervention planning across pharmacologic and psychosocial treatments.

## 1 Introduction

The central question in addiction is not why drugs are rewarding, or why habits form. It is why evidence stops changing behavior. Contemporary neuroscience supports opioid use disorder (OUD) as a brain-based disorder involving learning, motivation, and control systems (1, 2). However, in routine clinical care, these mechanistic insights are not always translated into a shared, practical framework that connects neurobiology with lived experience. Patients commonly describe phenomena such as craving, compulsion, narrowed attention or “tunnel vision,” and using opioids before conscious awareness or deliberate decision-making fully emerge. Clinicians often recognize these experiences in practice, yet they are not always integrated into a coherent explanatory model that can guide psychoeducation, relapse-prevention planning, medication-based treatment, and psychosocial interventions across different stages of recovery.

Current addiction neuroscience converges on four broad accounts of how drug use becomes entrenched, each grounded in well-characterized circuitry. Incentive-salience models explain how drug cues acquire disproportionate motivational pull through mesolimbic value processing (3, 4). Reinforcement-learning and habit models describe the shift from goal-directed use to stimulus-bound responding and, eventually, compulsion (5, 6), with recent transdiagnostic work situating compulsivity within shared circuit mechanisms (7). Allostatic and negative-reinforcement models show how withdrawal, stress, and dysphoria recalibrate motivation around relief seeking (8, 9). Salience-network and executive-control models place these dynamics within insula, anterior cingulate, and prefrontal systems that shape attention, conflict monitoring, and inhibitory control (10–12). Together, these accounts are complementary rather than competing. They illuminate different levels of the same clinical problem. What remains less developed is a shared computational frame that relates these mechanisms to one another and to the lived experience of relapse vulnerability, including craving, narrowed attention, automaticity, and the feeling of using before choice is fully available.

A unifying candidate comes from computational neuroscience and psychiatry: the brain as an active information-processing system that continuously predicts, updates, and selects actions under uncertainty. In predictive processing and active inference formulations, behavior reflects inference about hidden states and policies; learning depends on prediction errors and, critically, on precision weighting—the confidence assigned to priors versus incoming evidence (13–16). This language maps closely onto OUD relapse phenomena such as cue-bound certainty, narrowed action repertoires, and persistent overvaluation of opioid-related outcomes despite negative consequences, while also enabling computational and model-based approaches that complement symptom-based assessment (17–19).

Clinicians routinely recognize addiction-related rigidity: repetitive thinking, stereotyped responding, limited emotional range, and reduced tolerance of uncertainty without defaulting to opioid use. Information theory provides a useful translation layer here: adaptive cognition requires a balance between stability and flexibility—enough constraint to sustain coherent goals, but enough variability to explore alternatives and update beliefs (20, 21). Across theoretical and empirical work, entropy and complexity have been used to characterize the richness of neural and cognitive dynamics, with low-entropy states associated with rigidity and perseveration and higher complexity linked to psychological flexibility (22, 23). In OUD, relapse risk can be understood clinically as a collapse of flexibility under stress, cues, and withdrawal. EEG complexity, entropy, and microstate metrics have been reported to differ across substances and clinical states, with effect direction and specificity varying by methodology and longitudinal context (24–26).

OUD relapse is not only a disorder of reward and choice; it is also a disorder of awareness. Many patients relapse in ways that feel dissociated from reflective control: behavior unfolds before craving is clearly recognized, or craving is experienced as an engulfing state with limited mental space for alternatives, often with a strong interoceptive component—withdrawal, arousal, pain, dysphoria. Contemporary accounts of consciousness emphasize that conscious access depends on effective integration of information across distributed networks (27, 28), while interoceptive models highlight the role of bodily signal representation in self-regulation (29, 30). Disruption of insula-centered interoceptive and salience systems has been causally linked to craving and addiction persistence, with broader anterior cingulate involvement implicated in impaired control and integration, directly aligning neurobiology with clinical phenomenology (31–33).

We propose the Informational Dysregulation Framework of Addiction (IDFA) as a candidate clinical operationalization of relapse vulnerability in OUD—a disorder of informational balance across three interacting axes: precision (maladaptive certainty and policy canalization), entropy and flexibility (contraction of behavioral and representational space under stress, cues, and withdrawal), and awareness and integration (impaired interoception and conscious access). Although developed and illustrated here in OUD, the framework is pitched at the level of addiction broadly. OUD serves as the worked clinical instantiation because relapse vulnerability is particularly stark, the neurobiology is well characterized, and the treatment ecosystem—medications for opioid use disorder, contingency management, cognitive-behavioral therapy, and mindfulness-based relapse prevention—is sufficiently differentiated to allow IDFA’s intervention-mapping claims to be evaluated. The three axes are not specific to opioids. They are intended to apply across substance and behavioral addictions, with the relative dominance of each axis varying by substance, by individual, and by stage of recovery. Based on first principles, extension beyond OUD is expected. Substance-specific neurobiology will shape how this takes form, and the framework remains in its initial OUD-focused theoretical formulation. Recent work synthesizing the neural and computational basis of compulsivity across neuropsychiatric disorders (7) provides a complementary circuit-level account; IDFA proposes the computational dysregulation that compulsivity is one expression of. Whether this framing offers incremental clinical or scientific utility beyond existing models is the question the present paper develops.

## 2 Methods

### 2.1 Review approach

This study used structured integrative narrative synthesis to examine whether diverse literatures on addiction, information processing, and relapse could be organized into a coherent clinical framework. This approach was selected because the evidence base is methodologically heterogeneous, spanning theoretical formulations, human neuroimaging, lesion-network mapping, computational modeling, electrophysiological signal analysis, and clinical treatment trials. These sources differ in study design, measurement tradition, operational definitions, and level of analysis, making quantitative synthesis less suitable for the present theory-building aim. The review integrated literature from addiction neuroscience, computational psychiatry, predictive processing, information theory, neural complexity research, interoception, and clinical relapse prevention. The synthesis followed established guidance for narrative review (34) and translational theory construction in psychiatry (35), and was guided by the Scale for the Assessment of Narrative Review Articles (SANRA) (36). This synthesis was not designed as a systematic review, scoping review, or meta-analysis, and no formal risk-of-bias grading was performed; source quality was instead handled through the source-prioritization criteria described in Section 2.3.

### 2.2 Search strategy

We searched PubMed, PsycINFO, and Scopus for English-language literature published between January 2000 and March 2026, supplemented by Google Scholar for citation chaining. The January 2000 cutoff reflects the consolidation of functional neuroimaging, computational psychiatry, and contemporary neurobiological models of addiction during the late 1990s; selected foundational sources from earlier decades (Shannon, Wiener, Robinson and Berridge, Schultz et al.) were retained as canonical citations. Search strings combined controlled vocabulary and free-text terms across seven thematic clusters: predictive processing and active inference; precision weighting and dopaminergic prediction error; entropy, neural complexity, and EEG signal diversity; interoception, salience network, and insula; consciousness, global workspace, and integrated information theory; foundational addiction neurobiology and compulsivity; and treatment evidence for OUD relapse prevention. Reference lists of major reviews and foundational sources were hand-searched for additional citations.

### 2.3 Source selection and prioritization

Sources were prioritized according to an explicit hierarchy. Human computational modeling, neuroimaging with computational decomposition, and lesion-network studies were given primacy for mechanism claims. Systematic reviews and methodologically rigorous primary studies were prioritized for EEG and complexity claims, with directional heterogeneity in the literature acknowledged rather than adjudicated. Theoretical frameworks with strong empirical anchoring were prioritized for interoception, salience-network, and consciousness claims, with particular weight given to lesion-network evidence linking distributed insula–anterior cingulate circuitry to addiction remission. Established treatment evidence and contemporary consensus documents were prioritized for clinical translation. Animal-model studies were cited for theoretical and mechanistic context where they informed foundational computational constructs; clinical claims about opioid use disorder and relapse vulnerability were grounded in human studies, reviews, and treatment evidence.

### 2.4 Synthesis

Evidence was synthesized thematically across the three informational axes (precision, entropy and complexity, awareness and integration) and the self-reinforcing loop linking them. Theoretical and empirical claims were distinguished. Where empirical findings diverged across substances, methodologies, or clinical states—most notably in the EEG complexity literature—divergence was noted rather than resolved. This synthesis did not involve human or animal participants and did not require ethical approval. The full database search strategy, source selection criteria, evidence hierarchy, and SANRA self-assessment are provided in **Supplementary Tables S1**–**S3**.

## 3 Integration of the literatures: the IDFA model

**Figure 1:**
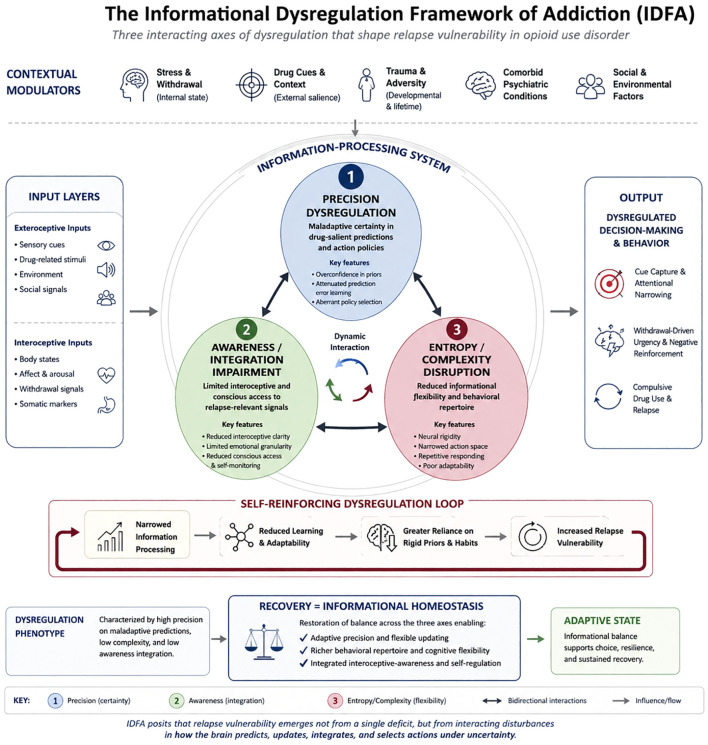
Informational Dysregulation Framework of Addiction (IDFA) applied to OUD relapse: three interacting informational axes. The schematic depicts OUD relapse vulnerability as dysregulation across three mutually interacting informational axes: precision dysregulation (maladaptive certainty assigned to opioid-salient predictions and policies), entropy and complexity collapse (reduced cognitive and behavioral flexibility under stress, cues, and withdrawal), and awareness and integration impairment (limited interoceptive and conscious access to relapse-relevant signals). The three axes are presented as overlapping rather than independent domains because clinical presentations typically express dominance on one axis with contributions from the others. Recovery is conceptualized as restoration of informational homeostasis across all three domains—reliable priors that remain open to revision, flexibility that does not collapse under stress, and bodily and reflective awareness that reaches consciousness early enough to inform policy selection.

The synthesis produced an information-processing model of relapse vulnerability in opioid use disorder, organized around three interacting domains and a self-reinforcing loop linking them. Fifty-five sources informed the model. These are organized by IDFA axis and sub-cluster in **Supplementary Table S4**, which presents the synthesis corpus together with brief description of how each source contributed to the model. These 55 sources directly informed model construction; additional references in the manuscript supported treatment translation, methodological framing, and publication-context claims. The axis-mapped key sources are summarized in **Table 1** and the overall architecture is illustrated in **Figure 1**. The three axes and the loop are developed in detail below.

**Table 1:** Selected key sources organized by IDFA construct domain.

Domain	Key sources (reference numbers)	Role in IDFA
Theoretical and information-theoretic foundations	Shannon (20); Wiener (21); Friston (15); Clark (14); Parr, Pezzulo & Friston (13); Friston, Stephan, Montague & Dolan (19)	Active inference, predictive processing, and information-theoretic frame underlying all three axes; brain as inference and policy-selection system under uncertainty
Precision dysregulation (stability axis)	Schultz, Dayan & Montague (37); Schwartenbeck et al. (17); Diederen & Fletcher (18); Daw, Niv & Dayan (38); Niv (39); Adams et al. (40); Haarsma et al. (69)	Dopaminergic encoding of prediction error and its precision; model-based/model-free reinforcement learning; precision and uncertainty parameters in computational psychiatry
Entropy and complexity collapse (flexibility axis)	Tononi & Edelman (41); Carhart-Harris et al. (22); Carhart-Harris (23); Deco & Kringelbach (42); Lempel & Ziv (44); Bandt & Pompe (45); Costa, Goldberger & Peng (46); von Wegner et al. (26); Bel-Bahar et al. (24); Liu et al. (25); Jui et al. (73); Diaz et al. (74)	Neural complexity foundations; the entropic brain framework; complexity-measurement toolkit (Lempel-Ziv, permutation entropy, multiscale entropy, microstate complexity); EEG correlates of substance use, abstinence, and recovery
Awareness and integration impairment (integration axis)	Dehaene, Lau & Kouider (27); Mashour et al. (28); Tononi & Koch (49); Oizumi, Albantakis & Tononi (50); Naqvi et al. (31); Naqvi & Bechara (56); Joutsa et al. (32); Fox (33); Khalsa et al. (51); Seth (53); Critchley et al. (30); Paulus & Stewart (29); Craig (52); Barrett, Quigley & Hamilton (54)	Global workspace and integrated information accounts of conscious access; insula and salience-network anchoring of interoception and addiction; lesion-network evidence that distributed insula–anterior cingulate circuitry is causally implicated in addiction remission

Sources are illustrative of the high-yield evidence anchoring each construct, not exhaustive; full citations appear in the reference list.

## 4 Precision dysregulation: the stability axis

Addiction is often described clinically as knowing better but doing the same thing. Patients report that drug-related predictions and action policies become rigid and resistant to disconfirming evidence: cues acquire disproportionate certainty, opioid-related outcomes are persistently overvalued, and the perceived space of available alternatives narrows. IDFA conceptualizes this as a disturbance in precision weighting—how strongly the brain treats certain predictions and policies as reliable, and therefore action-guiding. In predictive processing and active inference, the brain continuously infers hidden states and selects policies under uncertainty; learning depends not only on prediction errors but on how much confidence (precision) is assigned to them and to competing priors (13–16, 19).

A central mechanistic bridge between computation and clinical addiction is the dopaminergic encoding of prediction error and its precision. Phasic dopamine signaling tracks prediction error in a manner consistent with reinforcement learning (37), and subsequent work has shown that dopaminergic midbrain signaling tracks not only the magnitude but the expected precision of prediction errors (17, 18). This provides a biologically plausible pathway by which drug-related cues and learned values acquire disproportionate weight relative to competing goals, and by which the system increasingly privileges drug-salient predictions and policies over time (3, 4).

Precision dysregulation integrates naturally with established accounts of how addiction becomes entrenched. The actions-to-habits-to-compulsion trajectory describes how reinforcement learning and repeated drug-taking shift behavior from goal-directed choice to automated responding (5–7), a transition that can be formalized as a shift in the balance between model-based and model-free control (38, 39). IDFA clarifies that this shift is not merely behavioral but informational: maladaptive certainty becomes assigned to drug-salient inferences, reducing responsiveness to disconfirming evidence and narrowing the space of perceived alternatives. Computational psychiatry frameworks indicate that precision, learning rate, and uncertainty parameters can be specified, measured, and tracked across clinical states (19, 40), supporting the prospect of treating precision dysregulation as a clinically meaningful target rather than an abstract construct.

In clinical terms, precision dysregulation explains cue capture, perseverative relapse risk, and the patient experience of being pulled toward use despite insight. It also provides a coherent mechanistic rationale for interventions that reduce volatility in the decision environment, stabilize internal state, and enable re-learning over time. Precision dysregulation is therefore treated as one axis of relapse vulnerability, not as a complete account on its own. Specific intervention levers and predicted axis-linked treatment effects are developed in Section 9 and **Table 2**.

**Table 2:** Operational mapping of IDFA constructs to candidate neural and computational markers, clinical expression, example intervention levers, and conditions under which each axis would be falsified.

Construct	Candidate neural and computational markers	Clinical expression	Example intervention levers	Falsification conditions
Precision dysregulation (stability axis)	Aberrant precision-weighting of drug-salient prediction errors; biased policy selection on probabilistic reversal learning, two-step planning, and cue-primed decision tasks; altered model-based/model-free RL balance; cue-reactive mesolimbic and fronto-striatal signaling	Cue-bound craving; high-certainty rapid lapses; perseveration on opioid-seeking despite insight; narrowed perceived alternatives	MOUD (methadone, buprenorphine) to stabilize the decision context; contingency management to reshape reinforcement of non-use; cue-control planning; relapse-prevention rehearsal	Relapse risk does not track precision-related computational parameters on cue-primed decision tasks; effective treatments (MOUD, contingency management) do not normalize these parameters; cue-driven choice bias is more parsimoniously explained by response-inhibition deficits without invoking precision weighting
Entropy and complexity collapse (flexibility axis)	Reduced EEG signal diversity (Lempel-Ziv complexity, multiscale and permutation entropy, microstate-sequence complexity); reduced behavioral repertoire diversity on ecological momentary assessment of coping	Repetitive thinking; stereotyped routines; narrowed coping under stress, cues, or withdrawal; low tolerance of uncertainty without opioid use	CBT and DBT skills work; structured activity scheduling; graded exposure to uncertainty; behavioral coping rehearsal	EEG complexity and behavioral repertoire diversity do not differ between active-use and stable-recovery states; treatments that should expand the policy space (CBT, structured skills work) do not produce measurable change in flexibility metrics; clinical rigidity reduces without corresponding change in neural or behavioral diversity
Awareness and integration impairment (integration axis)	Reduced interoceptive accuracy on validated instruments (e.g., heartbeat discrimination, MAIA); increased craving-recognition latency in cue-exposure paradigms; lesion-network mapping to distributed insula–anterior cingulate circuitry	Using before noticing craving or withdrawal; low interoceptive clarity; craving experienced as engulfing or unnamed; dissociation between intention and action	Mindfulness-based relapse prevention; interoceptive training; biofeedback; meta-awareness practice integrated into psychotherapy	Interoceptive accuracy and craving-recognition latency do not predict relapse independently of cue exposure; awareness-oriented interventions (MBRP, interoceptive training) do not produce measurable change in these parameters; clinically defensible measures of integration cannot be operationalized at the patient level
Informational homeostasis (recovery target)	Coordinated normalization across precision-related computational parameters, neural and behavioral flexibility metrics, and interoceptive and metacognitive measures	Restored decision space; reliable priors that remain open to revision; flexibility that does not collapse under stress; bodily and reflective awareness that informs action selection before policy is committed	Combined and sequenced application of axis-targeted interventions according to dominant axis and stage of recovery; longitudinal measurement-based care	Sustained recovery is not associated with reversal of axis-specific markers; restoration of one axis does not propagate to the others; recovery outcomes are independent of any of the axis-linked measures

The mapping is illustrative rather than exhaustive; most patients present with mixed-axis dominance, and intervention sequencing reflects clinical context, recovery stage, patient preference, and treatment availability. RL, reinforcement learning; MAIA, Multidimensional Assessment of Interoceptive Awareness; MOUD, medications for opioid use disorder; CBT, cognitive behavioral therapy; DBT, dialectical behavior therapy; MBRP, mindfulness-based relapse prevention.

## 5 Entropy and complexity collapse: the flexibility axis

Clinicians recognize addiction-related rigidity as more than a behavioral habit: it includes repetitive thinking, stereotyped routines, narrowed affective range, and reduced tolerance of uncertainty without defaulting to opioid use. IDFA conceptualizes this as an entropy and complexity disturbance—a reduction in informational flexibility and diversity across neural and cognitive dynamics. Relapse, on this view, can be understood as informational bandwidth collapse: a contraction of behavioral and representational repertoires under stress, cue exposure, and withdrawal.

Information theory provides a useful translation layer. Foundational work in information theory and cybernetics established a language for describing how systems regulate, transmit, and stabilize information (20, 21). Applied to the brain, adaptive cognition requires a balance between stability and variability—enough structure to maintain coherent goals, but enough flexibility to explore alternatives and update beliefs (41, 42). The entropic brain framework links mental rigidity to constrained neural dynamics: overly constrained dynamics correspond to narrowed cognitive and experiential repertoires, while richer dynamics enable revision of entrenched models of self and world (22, 23). The link between neural complexity and consciousness is further developed in integrated-information accounts, which propose that informational states reaching conscious access require both sufficient differentiation and sufficient integration (43). IDFA does not treat higher entropy as automatically beneficial; the target is informational homeostasis, an adaptive middle ground between overly ordered and under-constrained states.

Electroencephalography offers a practical bridge between this conceptual axis and measurable correlates. Complexity and entropy of neural signals can be quantified using established methods including Lempel–Ziv complexity (44), permutation entropy (45), multiscale entropy (46), and microstate-sequence analysis (26), supporting a coherent measurement toolkit for this axis. Scoping and systematic reviews of EEG in substance use populations report alterations across spectral features, connectivity, and complexity-related measures, with ongoing investigation of how these track clinical state, relapse vulnerability, and recovery (24, 25). Effect direction varies across substances and methodologies, and translation from signal-level measures to patient-level meaning requires caution. The entropy/complexity axis is intended as a translational construct linking flexibility, repertoire diversity, and candidate neural signal measures; no single EEG entropy metric is treated as a direct biomarker of relapse risk.

Clinically, entropy and complexity collapse corresponds to reduced behavioral and cognitive flexibility, narrowed coping repertoires, and diminished capacity to generate non-opioid alternatives under stress or cue exposure. This contraction may present as rapid defaulting to previously reinforced opioid-seeking scripts under distress, impaired ability to generate alternative policies during cue exposure, and low tolerance of uncertainty without resorting to opioid use. Similar reductions in behavioral flexibility have been described across substance use disorders, supporting the transdiagnostic relevance of this axis while preserving its specific weight in opioid-related relapse contexts (47, 48).

## 6 Awareness and integration impairment: the integration axis

Patients commonly describe relapse as occurring before they realize they were choosing, or report craving as an engulfing state that crowds out alternative goals. IDFA treats this as an awareness and integration impairment: information relevant to self-regulation—interoceptive signals, bodily prediction errors, early craving cues—fails to become sufficiently integrated and consciously accessible to influence action selection in time. The clinical timing matters as much as the content: it is not only that signals are absent but that they arrive too late, or too fragmented, to redirect behavior before opioid use is initiated.

Contemporary theories of consciousness emphasize that conscious access depends on effective integration and broadcasting of information across distributed neural systems. Global workspace formulations describe consciousness as a functional state in which information becomes widely available for flexible cognition and control (27, 28), while integrated information approaches focus on the degree to which a system’s informational states are both differentiated and unified (49, 50). These frameworks supply a functional language for why some informational states are richly integrated while others remain fragmented or inaccessible. In clinical addiction, the relevant operationalization is timing and integration: latency to conscious recognition of craving or withdrawal cues, and whether corrective signals achieve sufficient integration to redirect behavior before opioid use is initiated.

Interoception provides the most clinically tractable bridge to this axis. Interoceptive signaling supplies the brain with information about bodily state—withdrawal, stress arousal, pain, dysphoria—that is essential for valuation, self-regulation, and decision-making (29, 30, 51, 52). Within active inference accounts, interoceptive prediction errors must achieve precision-weighted integration to influence policy selection (53–55). Paulus and Stewart’s framework positions interoceptive dysfunction as a mechanistic contributor to craving and maladaptive choice (29). Lesion-network evidence provides causal-strength support: Naqvi and colleagues showed that focal insular damage can disrupt cigarette addiction (31, 56), and Joutsa and colleagues subsequently demonstrated that lesions associated with addiction remission map to a distributed insula–anterior cingulate circuit rather than to a single anatomical site (32, 33). The implication is that intact awareness and integration circuitry is not merely correlated with addiction but is functionally necessary for the persistence of certain addictive drives.

In clinical terms, awareness and integration impairment explains why relapse can feel automatic, why craving can dominate attention before reflective control engages, and why interventions that strengthen interoceptive awareness and meta-awareness can restore decision space. Specific intervention levers and predicted axis-linked treatment effects are developed in Section 9 and **Table 2**.

## 7 The self-reinforcing neural–cognitive information loop

**Figure 2:**
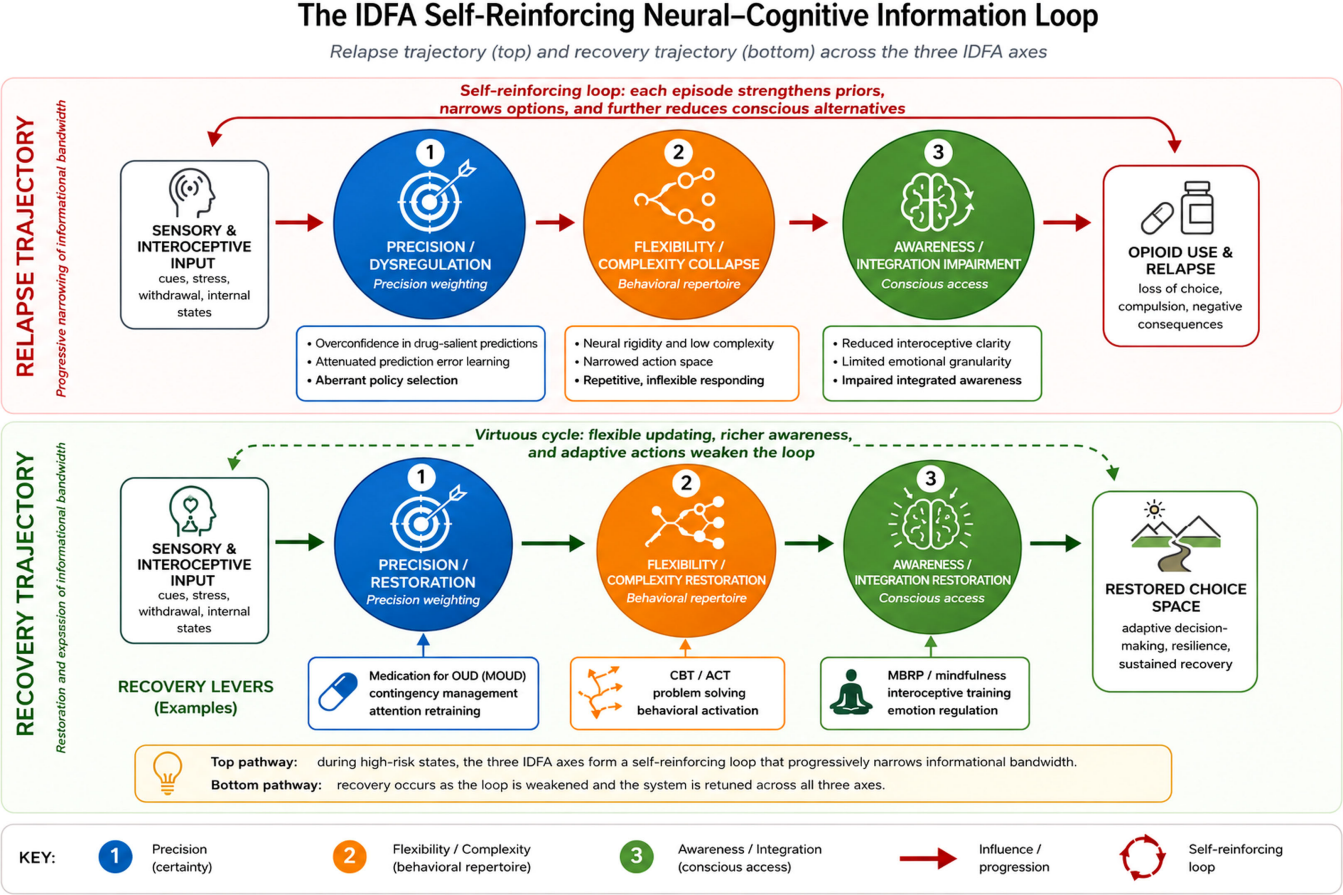
Self-reinforcing neural–cognitive information loop in OUD relapse and recovery. The diagram illustrates the dynamic loop linking the three IDFA axes during high-risk states and relapse trajectories. Biased prediction and rigid policy selection (precision dysregulation) narrow the behavioral and representational repertoire (entropy and complexity collapse), which in turn limits the integration of interoceptive and reflective signals into consciously accessible decision processes (awareness and integration impairment). Each episode of use strengthens the priors that generated it, further canalizes policy selection, and reduces the range of consciously accessible alternatives, closing the loop. Recovery is depicted as disruption of this loop through retuning of precision, restoration of adaptive entropy and complexity, and enhanced interoceptive and reflective awareness, with the loop opening rather than tightening across recovery trajectories.

The three axes do not operate independently. Precision dysregulation locks the system into drug-salient predictions and policies (13, 14, 16, 17); entropy and complexity collapse narrows behavioral and representational repertoires under stress, cues, and withdrawal (22, 23); and awareness and integration impairment prevents corrective prediction errors—including interoceptive signals—from reaching reflective control in time (29, 32, 53). Each axis exacerbates the others. Rigid priors reduce the system’s tolerance for variability, which in turn narrows the policy space and impoverishes the signals that might have prompted updating. Limited integration of bodily and reflective awareness allows drug-salient predictions to drive action before they can be revised. Over time these processes form a self-reinforcing loop: each episode of use strengthens the priors that generated it, further canalizes policy selection, and reduces the range of consciously accessible alternatives. The loop is depicted schematically in **Figure 2**.

Recovery, on this view, is restoration of informational homeostasis. Prediction errors regain the capacity to update beliefs, as precision is retuned across drug-salient and non-drug-salient inferences. Behavioral and cognitive flexibility is restored, as entropy and complexity move from collapse toward an adaptive middle ground. Interoceptive and reflective awareness re-engage with action selection, restoring decision space before relapse trajectories close. This is not a return to a pre-addicted baseline but an active rebalancing—reliable priors that remain open to revision, flexibility that does not collapse under stress, and bodily and reflective awareness that reaches consciousness early enough to inform policy selection. Because the axes mutually reinforce through the loop, intervention at any axis can weaken the loop and propagate improvement to the others; this is what makes axis-targeted entry points clinically tractable rather than arbitrary. The framework’s empirical predictions, intervention-mapping claims, and clinical applications are developed in Section 9.

## 8 Cross-level integration: existing addiction accounts as expressions of informational dysregulation

**Figure 3:**
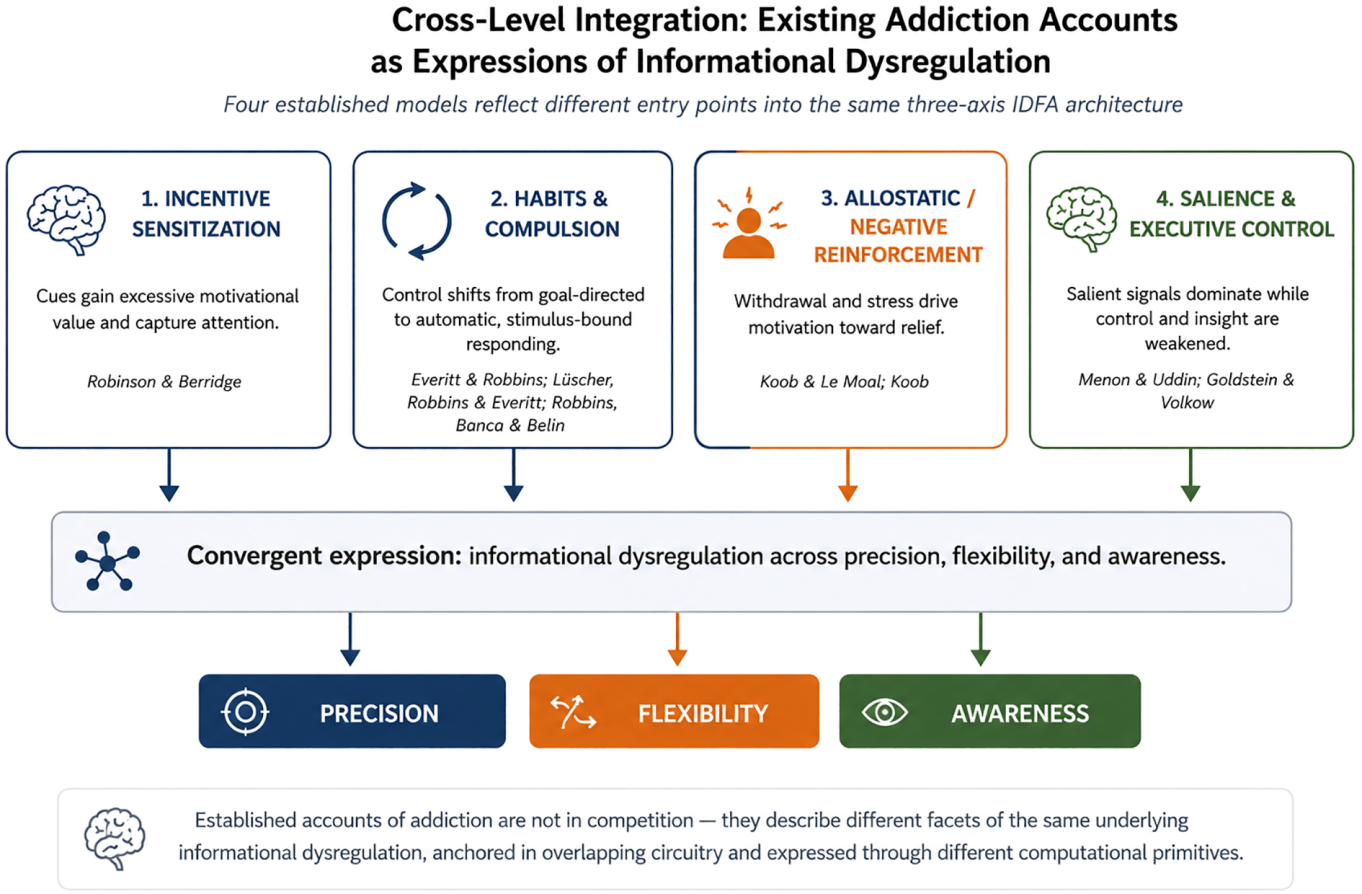
Cross-level integration map: existing addiction accounts as coupled expressions of informational dysregulation. The three-column architecture moves from circuit-level mechanism to computational construct to clinical phenomenon. Each row shows how an established account of addiction—incentive sensitization, the actions-to-habits-to-compulsion trajectory, allostatic and negative-reinforcement processes, and salience-network reactivity with executive-control disruption—locates within IDFA’s three-axis frame. Read in this way, the dominant accounts are not in competition but describe different facets of the same underlying informational dysregulation, anchored in overlapping circuitry, expressed through different computational primitives, and presenting through different but related clinical phenomena. The map provides the conceptual basis for the claim that diverse interventions can each reduce relapse risk because they act on different axes of the same underlying dysregulation rather than competing for a single mechanistic target.

The three axes provide an organizing layer at which the dominant accounts of addiction become visible as coupled expressions of the same underlying dysregulation rather than competing explanations. **Figure 3** maps the cross-level architecture: each column moves from circuit-level mechanism to computational construct to clinical phenomenon, and each row shows how an existing account locates within IDFA’s three-axis frame.

Incentive sensitization (3, 4) is expressed as precision dysregulation in mesolimbic value circuitry. Drug-related cues acquire disproportionate motivational pull through aberrant precision weighting of drug-salient predictions, presenting clinically as cue-bound wanting, craving, and the felt urgency of approach behavior. The axis-level location is precision; the contributing circuit is mesolimbic; the clinical phenomenon is cue-driven craving.

The actions-to-habits-to-compulsion trajectory (5–7, 57) is expressed as precision dysregulation in fronto-striatal policy selection, with progressive narrowing of the policy space as habit and compulsion consolidate. Maladaptive certainty becomes attached to drug-salient inferences, while the diversity of available non-drug policies contracts. The clinical phenomena are automaticity, perseveration, reduced goal-directed control, and the experience of acting before reflection.

Allostatic and negative-reinforcement processes (8, 9) are expressed as a combination of precision dysregulation and entropy and complexity collapse. The system over-weights withdrawal-relief expectations, while narrowing coping diversity under dysphoric load—producing the clinical phenomena of withdrawal-driven urgency, stress-induced relapse, and the contraction of non-opioid coping repertoires.

Salience-network reactivity and executive-control disruption (10–12, 15, 58) are expressed primarily as awareness and integration impairment along the insula–anterior cingulate–prefrontal axis. The clinical phenomena are impaired insight under craving, using before noticing, and dissociation between intention and action—the same phenomenology IDFA’s awareness axis was developed to explain.

Read in this way, the dominant accounts are not in competition. They describe different facets of the same underlying informational dysregulation, anchored in overlapping circuitry, expressed through different computational primitives, and presenting through different but related clinical phenomena. Among other implications, this clarifies why diverse interventions can each reduce relapse risk: they act on different axes of the same underlying dysregulation rather than competing for a single mechanistic target. The mapping of specific interventions to specific axes is developed in Section 9 and **Table 2**.

## 9 Discussion

### 9.1 Summary

IDFA reframes relapse vulnerability in OUD as dysregulated information processing: an imbalance in how the brain predicts, updates, and integrates experience under uncertainty. It is positioned as an integrative layer rather than a replacement for established neurobiological models of addiction (1, 2, 59). Its contribution is to link reinforcement learning, habit formation, compulsivity, allostasis, salience, and executive control to clinically recognizable experiences such as cue capture, withdrawal-driven urgency, narrowed awareness, and recurrent relapse risk. The framework is organized around three interacting axes: precision dysregulation, entropy and complexity collapse, and awareness and integration impairment (**Figure 1**). Together, these axes form a self-reinforcing loop that narrows informational bandwidth and policy flexibility during high-risk states and relapse trajectories (**Figure 2**). Existing addiction accounts can therefore be mapped onto IDFA’s cross-level architecture (**Figure 3**) as coupled expressions of relapse vulnerability rather than as competing explanations.

### 9.2 Translational and clinical implications

IDFA is designed to support clinical formulation, treatment planning, and measurement-based relapse prevention across heterogeneous OUD presentations. Rather than proposing a new diagnostic category, it offers a mechanism-linked map for identifying which processes may be most active in a given relapse context. Different interventions can then be understood as acting on distinct informational axes, often in combination and at different stages of recovery. Medications for OUD, particularly methadone and buprenorphine, stabilize withdrawal physiology and the decision context, with established benefits for retention and mortality (60, 61). Within IDFA, this stabilization may reduce cue-driven volatility and the maladaptive certainty assigned to opioid use as the dominant action policy. Contingency management provides reliable, salient reinforcement for non-use and can be interpreted as reshaping learning and precision weighting around alternative outcomes (62, 63). Cognitive-behavioral therapy may expand the policy space by strengthening coping repertoires and behavioral flexibility under stress and cue exposure (64). Mindfulness-based relapse prevention and related awareness-oriented approaches may act on the integration axis by improving interoceptive clarity and earlier detection of craving, withdrawal, and affective cues, with randomized evidence supporting relapse reduction compared with standard care (65). **Table 2** links IDFA constructs to candidate neural markers, computational analogs, clinical phenomena, intervention levers, and falsification conditions. These mappings are hypothesis-generating rather than prescriptive. Most patients will show mixed-axis dominance, and intervention sequencing should reflect clinical context, recovery stage, patient preference, and treatment availability. The goal is to make the mechanistic rationale for relapse-prevention practice explicit, clinically usable, and testable.

### 9.3 IDFA-guided clinical workflow for OUD relapse prevention

IDFA can be applied at intake and during relapse-prevention follow-up through a four-step loop. The workflow is intended as a formulation aid rather than a prescriptive algorithm, and mixed-axis presentations are expected rather than exceptional. Clinicians retain judgment over how heavily to weight each step in a given encounter.

Step A. Assess today’s relapse context and state.

Withdrawal, negative affect, and interoceptive load (pain, dysphoria, arousal); cue exposure and access (people, places, paraphernalia, money, availability); stress, sleep, comorbid anxiety, depression, and trauma reminders; current medications, including medications for opioid use disorder (MOUD) adherence and dose stability, and recent lapses; protective factors and constraints (supports, structure, contingencies).

Step B. Identify the dominant IDFA axis and a secondary axis.

Precision-dominant: cue capture, rapid auto-pilot use, strong certainty that opioids are the only workable solution. Flexibility-dominant: narrowed coping repertoire, repetitive routines, low tolerance of uncertainty, limited alternatives under stress. Awareness-dominant: using before noticing, low interoceptive clarity, craving and withdrawal signals experienced as engulfing or unnamed.

Step C. Match interventions to the dominant axis.

Precision-focused: stabilize the decision context and reduce cue-driven volatility (optimize MOUD; contingency management; cue-control planning). Flexibility-focused: expand behavioral options (skills-based psychotherapy including CBT and DBT skills; structured activity scheduling; graded exposure to uncertainty). Awareness-focused: improve early detection and integration of craving and withdrawal signals (mindfulness-based relapse prevention [MBRP]; interoceptive training; biofeedback).

Step D. Track metrics and iterate.

Precision: cue-induced craving ratings; lapse frequency; time-to-first-use in high-risk contexts. Flexibility: number and diversity of non-opioid coping actions used per week; activity-scheduling adherence. Awareness: latency to noticing craving and withdrawal; interoceptive clarity ratings; mindfulness or interoceptive practice minutes.

The workflow is intentionally simple and does not require new instruments. The metrics are intended to be tracked qualitatively or with brief self-report scales as part of routine clinical contact, with axis emphasis updated as the patient’s stage and context change.

### 9.4 Clinical vignettes: same DSM diagnosis, different IDFA profiles

Patient A (precision-dominant relapse). A 32-year-old with OUD reports rapid lapses after passing a familiar pharmacy and encountering an old using contact. He describes a sudden, high-certainty “I’m already going to use” feeling with minimal deliberation. *IDFA profile:* primary precision dysregulation (cue-weighted policies) with secondary flexibility narrowing. *Treatment emphasis:* prioritize stability and learning levers: optimize MOUD and adherence supports; add cue-control and contingency strategies; rehearse a rapid-response plan for high-risk contexts. *Monitoring focus:* cue-induced craving intensity and lapse frequency in identified trigger situations.

Patient B (awareness/flexibility-dominant relapse). A 32-year-old with OUD reports using before realizing she is craving, especially during early withdrawal, insomnia, and interpersonal stress. She struggles to label bodily states and notices craving only when it becomes overwhelming. *IDFA profile:* primary awareness and integration impairment with secondary entropy and complexity collapse (rigid coping under stress). *Treatment emphasis:* prioritize awareness and flexibility levers: interoceptive labeling and early-warning tracking; MBRP; structured coping rehearsal and activity scheduling; stabilize withdrawal physiology with MOUD optimization and sleep and pain supports. *Monitoring focus:* latency to noticing craving and withdrawal cues, and weekly number and diversity of non-opioid coping actions used.

The two patients share a DSM diagnosis and would qualify for the same broad treatment guideline. Their IDFA profiles differ, and so does the rational emphasis of their treatment plans. The framework’s clinical value rests precisely on this kind of disaggregation: helping clinicians identify which mechanism is most active for a given patient in a given context, and matching the intervention to it.

### 9.5 What this changes in practice

Six practical implications follow.

First, an IDFA axis profile (primary and secondary) for OUD relapse risk can be written into the clinical assessment alongside the DSM diagnosis, providing a mechanism-linked complement to symptom-based classification.

Second, at least one intervention lever targeting the dominant axis (**Table 2**) is selected for the treatment plan, with the mechanistic rationale named explicitly. This makes the link between mechanism and intervention visible to the patient, the clinician, and the team.

Third, one simple metric per axis is tracked over time to guide iteration: craving and cue-context ratings for precision, coping diversity for flexibility, and awareness latency for integration. The metrics are intentionally tractable in routine care.

Fourth, the axis profile supports non-moral explanation of relapse (informational dysregulation, not character flaw) and supports shared decision-making about which intervention to try, in what order, and with what monitoring target. Similar interpretive approaches have also been proposed in correctional OUD assessment, where variability or inconsistency in patient responses may itself carry clinically meaningful information rather than being treated reflexively as evidence of dishonesty (66).

Fifth, the dominant axis is reassessed after each lapse or relapse, and the emphasis is shifted as the patient’s stage and context change. Early recovery may emphasize precision and entropy levers; later recovery may shift weight toward awareness, integration, and recovery-capital building.

Sixth, comorbidity is treated as axis modulation rather than as confound. OUD rarely presents in isolation, and comorbid depression, anxiety, PTSD, and chronic pain are the clinical norm rather than the exception. Within IDFA, these conditions are understood to load the same three axes rather than to act as separate processes: depression and chronic pain typically load the awareness and precision axes through interoceptive blunting and pain-prior weighting; anxiety loads the precision axis through threat-prior heightening and policy-space narrowing; PTSD loads all three through intrusive priors, freeze-flight policy collapse, and dissociative awareness disruption. The framework accommodates these patterns by adjusting the axis profile to reflect the comorbid load rather than abstracting from it, which is consistent with the workflow’s emphasis on formulation over diagnosis.

These shifts are modest in operational terms and do not require new instruments or restructured services. The change is in how clinicians and patients talk about why relapse happened and which lever to pull next.

### 9.6 Intervention-mapped predictions

IDFA generates testable, intervention-mapped predictions for relapse vulnerability and recovery in OUD. The predictions are organized by axis; each names the expected direction of change in a measurable parameter, and the intervention through which the change is predicted to occur.

Precision axis. Higher relapse risk is predicted to be associated with more rigid, cue-biased policy selection under uncertainty on computational decision tasks—probabilistic reversal learning, two-step planning, and cue-primed decision tasks—reflected in higher inverse temperature, reduced exploration, and biased precision-weighting of drug-salient prediction errors (67, 68). Effective treatment for the precision axis (MOUD, contingency management) is predicted to reduce cue-driven choice bias and normalize these computational parameters over time, with changes in cue reactivity and craving mediating the reduction in relapse frequency (69). Computational neuroimaging and model-based behavioral assays support the feasibility of tracking these parameters at the individual-patient level across treatment course (70–72).

Flexibility axis. Lower neural and behavioral flexibility is predicted to predict relapse, operationalized through reduced EEG signal diversity—Lempel–Ziv complexity, multiscale and permutation entropy, and microstate-sequence complexity (44–46, 73, 74)—and through reduced real-world repertoire diversity, measurable by ecological momentary assessment of coping behaviors. Recovery is predicted to be associated with increased neural complexity and increased behavioral coping diversity, with changes in flexibility metrics mediating improvement in clinical outcomes that reflect this axis (rigidity, perseveration, narrowed coping under stress).

Awareness axis. Interoceptive and metacognitive deficits are predicted to predict relapse, measurable through validated interoceptive awareness and accuracy instruments and through craving-recognition latency in cue-exposure paradigms. Awareness-oriented interventions (MBRP, interoceptive training, biofeedback) are predicted to improve interoceptive clarity and earlier detection of urge states, with these changes mediating reductions in relapse frequency and severity.

A stronger test of the unified framework is longitudinal and multimodal: in a cohort followed across stages of recovery, sustained recovery should be associated with axis-linked normalization across all three measurable channels (precision-related computational parameters, neural and behavioral flexibility metrics, and interoceptive and metacognitive awareness).

### 9.7 Limitations

Several limitations qualify IDFA as presented here. First, the framework remains theoretical and has not yet been tested against patient-level longitudinal data. Its proposed clinical utility therefore remains provisional until multimodal OUD studies determine whether axis-linked markers predict relapse risk, track recovery trajectories, or respond to axis-targeted interventions in the directions hypothesized. The conclusions presented here should be interpreted as an integrative model grounded in high-yield evidence rather than an exhaustive accounting of all studies. In addition, several constructs central to the framework (including precision weighting, neural complexity, interoceptive processing, and awareness-related dynamics) remain methodologically heterogeneous across disciplines, with entropy and complexity metrics and consciousness frameworks varying in definitions and measurement assumptions. Computational task parameters, EEG complexity indices, microstate measures, and interoceptive scales are not yet standardized into a common patient-level reporting structure, and validated “IDFA axis scores” do not currently exist for routine clinical use. Transdiagnostic extension of the framework beyond OUD requires parallel theoretical and empirical development, since substance-specific neurobiology—particularly in interoceptive signaling and withdrawal phenomenology—may shift the relative weight of each axis. Translation from computational and signal-level measures to patient-level meaning should therefore remain cautious (22–25, 28, 75).

IDFA also does not exclude alternative explanations grounded in classical cognitive and learning models, including priming, habit formation, context effects, or reinforcement-based accounts. Many relapse-relevant phenomena can be described within these frameworks at the level of individual mechanisms. The intended contribution of IDFA is not to replace such accounts, but to organize them within a unified information-processing architecture that integrates precision, flexibility, and awareness dimensions. Empirical work will be required to determine whether this integrative formulation offers incremental predictive or clinical utility beyond existing models.

## 10 Conclusion

IDFA provides a clinically usable framework that integrates computational neuroscience, information theory, and awareness/interoception science into a coherent model of addiction and recovery. It reframes relapse vulnerability in opioid use disorder as a disorder of informational balance—how the brain predicts, updates, and integrates experience under uncertainty. Rather than reducing OUD to a single mechanism, IDFA organizes relapse risk across three interacting clinical axes: precision dysregulation (maladaptive certainty and rigid opioid-seeking policies), entropy/complexity disruption (collapsed flexibility and narrowed coping repertoires under stress or withdrawal), and awareness/integration impairment (delayed interoceptive and conscious access to relapse-relevant signals). Together, these processes help explain a core clinical paradox in OUD: cue-bound, high-certainty opioid use that can recur despite insight and escalating harm (1, 5, 13, 14, 19, 29, 31).

Clinically, IDFA offers a practical relapse-prevention lens. Clinicians can identify the dominant informational axis in a given relapse context, align interventions (e.g., medications for OUD, contingency management, skills-based psychotherapy, mindfulness/interoceptive training) with plausible mechanisms, and track simple axis-linked metrics over time. The framework also supports a measurement-informed future in which computational parameters, EEG complexity indices, and interoceptive markers complement symptom-based monitoring, without implying that any single biomarker is diagnostic (22–26, 29, 31, 71–74).

IDFA is intended to generate testable predictions and clinically useful hypotheses regarding relapse and recovery trajectories. Future longitudinal and multimodal research can examine whether restoration of precision balance, flexibility/complexity, and awareness/integration precedes and predicts sustained recovery under real-world relapse risk.

## Data Availability

The original contributions presented in the study are included in the article/**Supplementary Material**. Further inquiries can be directed to the corresponding author.
