# Supplementary material for: The informational dysregulation framework of addiction (IDFA): an information-processing model of relapse in opioid use disorder
Source: Front Psychiatry. 2026 Jul 13;17:1819543. doi: 10.3389/fpsyt.2026.1819543 (PMC13403627; doi:10.3389/fpsyt.2026.1819543)
Supplement: Supplementary file 1 [file Table1.docx]

**Supplementary Materials**

*The Informational Dysregulation Framework of Addiction (IDFA): An Information-Processing Model of Relapse in Opioid Use Disorder*

**Ovie Martin Albert** | Correspondence: ovie.albert1@ucalgary.ca

# **Supplementary Table S1. Full database-specific search strategy**

Searches were conducted across PubMed, PsycINFO, and Scopus for English-language literature published between January 2000 and March 2026. Google Scholar was used for citation chaining only, not for primary identification. Reference lists of major reviews and foundational sources were hand-searched. The search was conducted by the corresponding author. Search strings are presented in standardized Boolean syntax; database-specific MeSH and APA Thesaurus mappings were applied as appropriate. Foundational pre-2000 sources (Shannon 1948; Wiener 1961; Lempel & Ziv 1976; Robinson & Berridge 1993; Higgins et al. 1994; Schultz, Dayan & Montague 1997; Tononi & Edelman 1998) were retained as canonical citations identified through hand-searching of reference lists.

**Cluster 1 — Predictive processing and active inference in addiction**

("predictive processing" OR "active inference" OR "free energy principle" OR "Bayesian brain") AND ("addiction" OR "substance use" OR "opioid use disorder" OR "drug dependence" OR "relapse")

**Cluster 2 — Precision weighting and dopaminergic prediction error**

("precision weighting" OR "precision-weighted" OR "prediction error" OR "reward prediction error" OR "expected uncertainty" OR "model-based" OR "model-free" OR "reinforcement learning") AND ("dopamine" OR "dopaminergic" OR "mesolimbic" OR "fronto-striatal") AND ("addiction" OR "substance use" OR "opioid")

**Cluster 3 — Entropy, neural complexity, and EEG signal diversity in substance use**

("entropy" OR "Lempel-Ziv" OR "permutation entropy" OR "multiscale entropy" OR "approximate entropy" OR "sample entropy" OR "neural complexity" OR "signal diversity" OR "microstate") AND ("EEG" OR "electroencephalography" OR "electroencephalogram" OR "neural dynamics") AND ("substance use" OR "addiction" OR "opioid" OR "cocaine" OR "alcohol" OR "stimulant" OR "abstinence" OR "relapse")

**Cluster 4 — Interoception, salience network, and insula in addiction**

("interoception" OR "interoceptive" OR "insula" OR "insular cortex" OR "salience network" OR "anterior cingulate") AND ("addiction" OR "substance use" OR "opioid" OR "craving" OR "withdrawal" OR "relapse")

**Cluster 5 — Consciousness, global workspace, and integrated information theory**

("global workspace" OR "global neuronal workspace" OR "integrated information theory" OR "IIT" OR "phi" OR "conscious access" OR "consciousness") AND ("addiction" OR "substance use" OR "craving" OR "interoception" OR "salience")

**Cluster 6 — Foundational addiction neurobiology and compulsivity**

("addiction neurobiology" OR "brain disease model" OR "incentive sensitization" OR "incentive salience" OR "habit formation" OR "compulsion" OR "compulsivity" OR "allostasis" OR "negative reinforcement") AND ("substance use" OR "opioid use disorder" OR "drug dependence")

**Cluster 7 — Treatment evidence for OUD relapse prevention**

("opioid use disorder" OR "opioid dependence" OR "opioid addiction") AND ("methadone" OR "buprenorphine" OR "naltrexone" OR "medications for opioid use disorder" OR "MOUD" OR "opioid agonist treatment" OR "contingency management" OR "cognitive behavioral therapy" OR "mindfulness-based relapse prevention" OR "MBRP" OR "relapse prevention")

**Limits applied across all clusters:** English language; January 1, 2000 to March 31, 2026; human subjects (except for Clusters 1, 2, and 3, where computational and theoretical sources without human-subject limits were also included).

# **Supplementary Table S2. Source selection and evidence hierarchy**

Sources were prioritized according to the claim type each was used to support. The hierarchy below was applied across all seven thematic clusters.

| **Claim type** | **Primary sources (highest priority)** | **Secondary sources** | **Used with caution or excluded** |
| --- | --- | --- | --- |
| **Mechanism claims (how the brain implements a process relevant to IDFA)** | Human computational modeling; neuroimaging with computational decomposition; lesion-network studies | Pharmacological challenge studies in humans; targeted neuropsychological studies | Preclinical (rodent) extrapolation alone; mechanism claims based on theoretical reasoning unsupported by human data |
| **EEG and complexity claims** | Systematic reviews; methodologically rigorous primary studies with adequate sample sizes; replicated findings across populations | Small primary studies with consistent methodology | Single-study findings without replication; studies with substantial methodological variance |
| **Interoception, salience-network, and consciousness claims** | Theoretical frameworks with strong empirical anchoring; lesion-network evidence; consensus documents (e.g., Khalsa et al. interoception roadmap) | Targeted neuroimaging studies in addiction populations; psychometric instrument development studies | Frameworks without empirical anchoring or with contested measurement assumptions, used only with explicit acknowledgment of limitations |
| **Clinical translation claims** | Established treatment evidence (Cochrane reviews; large RCTs; well-powered cohort meta-analyses); contemporary consensus documents and clinical practice guidelines | Single-center RCTs with adequate methodology; observational cohort studies | Case series; expert opinion unsupported by trial evidence |

**Directional heterogeneity in the EEG complexity literature was acknowledged rather than adjudicated.** Where empirical findings diverged across substances, methodologies, or clinical states, the divergence was noted in the synthesis (Section 5 and Section 9.6) and reflected in the cautious framing of axis-linked predictions. Animal-model studies were cited for theoretical and mechanistic context where they informed foundational computational constructs; clinical claims about opioid use disorder and relapse vulnerability were grounded in human studies, reviews, and treatment evidence.

# **Supplementary Table S3. SANRA self-assessment**

The Scale for the Assessment of Narrative Review Articles (SANRA; Baethge, Goldbeck-Wood & Mertens, 2019) is a six-item instrument with each item scored 0 (low), 1 (moderate), or 2 (high). Possible total: 12. The self-assessment below was completed by the corresponding author.

| **SANRA item** | **Score** | **Justification** |
| --- | --- | --- |
| 1. Justification of the article’s importance for the readership | **2** | The Introduction establishes the clinical problem (relapse vulnerability in OUD), the gap in unifying clinical-mechanistic frameworks, and the relevance for clinicians and researchers in addiction medicine, computational psychiatry, and neuroscience. |
| 2. Statement of concrete aims or formulation of questions | **2** | The aim of proposing IDFA as a candidate clinical operationalization of relapse vulnerability is explicitly stated in the Introduction; the question of whether the framework offers incremental clinical or scientific utility beyond existing models is named as the question the paper develops. |
| 3. Description of the literature search | **2** | The Methods section names databases searched, date range, foundational pre-2000 inclusions, seven thematic clusters, and reference-list hand-searching. Full database-specific search syntax is provided in Supplementary Table S1. |
| 4. Referencing | **2** | All claims are anchored in cited sources; references are presented in Vancouver style; verified citations only. The reference list includes the full bibliography and is organized by order of first appearance. |
| 5. Scientific reasoning | **2** | The framework is developed through structured integrative synthesis with explicit reasoning at each stage. Theoretical and empirical claims are distinguished throughout. Where empirical findings diverge across substances, methodologies, or clinical states, divergence is noted rather than resolved. Limitations are explicit (Section 9.7). |
| 6. Appropriate presentation of data | **2** | Figures and tables present the framework’s core architecture (Figure 1), the self-reinforcing loop (Figure 2), and the cross-level integration map (Figure 3). Table 1 maps the literature to constructs; Table 2 provides operational mapping of constructs to candidate markers, clinical expression, intervention levers, and falsification conditions. |
| **TOTAL** | **12 of 12** |  |

***Note.*** *The self-assessment is provided in accordance with SANRA’s intended use as a methodological-quality framework for narrative reviews. Self-assessment scores are necessarily interpreted in the context of independent reviewer evaluation.*
