# Supplementary material for: The informational dysregulation framework of addiction (IDFA): an information-processing model of relapse in opioid use disorder
Source: Front Psychiatry. 2026 Jul 13;17:1819543. doi: 10.3389/fpsyt.2026.1819543 (PMC13403627; doi:10.3389/fpsyt.2026.1819543)
Supplement: Supplementary file 2 [file Table2.docx]

# Supplementary Table S4

*Included studies informing the Informational Dysregulation Framework of Addiction (IDFA): synthesis corpus organized by IDFA axis and sub-cluster.*

This supplementary table presents the 55 sources that directly informed the structured integrative narrative synthesis underlying the IDFA model. Sources are grouped by the primary IDFA axis to which each contributes, with sub-cluster designations indicating the thematic role of each source within its axis.

| **Ref #** | **Author(s) and year** | **Sub-cluster** | **Specific contribution to the IDFA model** |
| --- | --- | --- | --- |
| **Cross-cutting theoretical foundations (n = 9)** | | | |
| 13 | Parr, Pezzulo & Friston 2022 | Predictive processing foundations | Active inference framework; brain as inferential system under uncertainty |
| 14 | Clark 2016 | Predictive processing foundations | Predictive processing as embodied prediction; foundational scaffolding |
| 15 | Friston 2010 | Predictive processing foundations | Free-energy principle as unified brain theory |
| 16 | Miller, Kiverstein & Rietveld 2020 | Predictive processing in addiction | First developed predictive-processing account of addiction |
| 19 | Friston, Stephan, Montague & Dolan 2014 | Computational psychiatry | Computational psychiatry as a discipline; precision-related model parameters |
| 21 | Wiener 1961 | Cybernetics and information theory | Cybernetic foundations of biological information regulation |
| 67 | Pezzulo, Rigoli & Friston 2015 | Active inference and homeostasis | Active inference, homeostatic regulation, and adaptive behavioural control |
| 68 | Smith, Badcock & Friston 2021 | Computational psychiatry | Predictive coding and active inference in clinical neuroscience |
| 70 | Stephan et al. 2017 | Computational psychiatry | Computational neuroimaging strategies for single-patient predictions |
| **Precision dysregulation (the stability axis) (n = 11)** | | | |
| 3 | Robinson & Berridge 1993 | Incentive sensitization | Incentive-sensitization theory; cue-driven motivational capture |
| 4 | Berridge & Robinson 2016 | Incentive sensitization | Updated incentive-sensitization; wanting vs. liking distinction |
| 8 | Koob & Le Moal 2008 | Allostatic / negative reinforcement | Antireward system; allostatic load in chronic use |
| 9 | Koob 2013 | Allostatic / negative reinforcement | Negative reinforcement; withdrawal-driven motivation |
| 17 | Schwartenbeck et al. 2015 | Dopaminergic prediction error and precision | Dopaminergic midbrain encodes expected precision of prediction errors |
| 18 | Haarsma et al. 2021 | Dopaminergic prediction error and precision | Dopamine and prediction error; contemporary synthesis |
| 37 | Schultz, Dayan & Montague 1997 | Dopaminergic prediction error and precision | Foundational dopamine RPE signalling work |
| 39 | Niv 2009 | Reinforcement learning | Reinforcement learning in the brain; computational framework |
| 40 | Adams, Stephan, Brown, Frith & Friston 2013 | Precision weighting in psychiatry | Computational anatomy of psychiatric disorders; precision weighting |
| 69 | Haarsma, Marques, Uddin et al. 2020 | Dopaminergic prediction error and precision | Precision-weighting of unsigned prediction errors modulated by dopamine |
| 72 | Mollick & Kober 2020 | Computational models of addiction | Computational models of drug use and addiction |
| **Precision–flexibility coupling (n = 4)** | | | |
| 5 | Everitt & Robbins 2005 | Habits-to-compulsion trajectory | Actions-to-habits-to-compulsion trajectory |
| 6 | Lüscher, Robbins & Everitt 2020 | Habits-to-compulsion trajectory | Transition to compulsion; circuit-level mechanisms |
| 38 | Daw, Niv & Dayan 2005 | Model-based vs. model-free RL | Model-based vs. model-free RL; uncertainty-based competition |
| 71 | Fiore, Gu, Vacca et al. 2023 | Computational models of addiction | Computational models of behavioural addictions |
| **Entropy and complexity collapse (the flexibility axis) (n = 12)** | | | |
| 20 | Shannon 1948 | Information theory foundations | Foundational information-theoretic definition of entropy |
| 22 | Carhart-Harris et al. 2014 | Entropic brain framework | Entropic brain framework; conscious states and neural complexity |
| 23 | Carhart-Harris 2018 | Entropic brain framework | Entropic brain revisited; refinement of original theory |
| 24 | Bel-Bahar et al. 2022 | EEG in substance use disorder | EEG markers in substance use disorder treatment |
| 25 | Liu, Huang & Zhao 2022 | EEG in substance use disorder | Resting-state EEG, substance use, and abstinence |
| 26 | von Wegner, Luo & Laufs 2023 | Complexity measurement toolkit | Complexity measures for EEG microstate sequences |
| 42 | Deco & Kringelbach 2017 | Neural complexity and information processing | Hierarchy of information processing; intrinsic ignition framework |
| 44 | Lempel & Ziv 1976 | Complexity measurement toolkit | Lempel–Ziv complexity measure |
| 45 | Bandt & Pompe 2002 | Complexity measurement toolkit | Permutation entropy for time series |
| 46 | Costa, Goldberger & Peng 2005 | Complexity measurement toolkit | Multiscale entropy analysis of physiological signals |
| 73 | Ullah, Jui & Deo 2023 | EEG entropy methodology | EEG entropy for neurological disorder detection |
| 74 | Zunino, Pérez, Martín et al. 2017 | Complexity measurement toolkit | Complexity–entropy maps for EEG characterization |
| **Flexibility–awareness coupling (n = 2)** | | | |
| 41 | Tononi & Edelman 1998 | Neural complexity and consciousness | Consciousness and complexity; foundational neural complexity theory |
| 43 | Popiel, Koch & Tononi 2020 | Integrated information and complexity | Integrated information, complexity, and consciousness |
| **Awareness and integration impairment (the integration axis) (n = 17)** | | | |
| 10 | Menon & Uddin 2010 | Salience network | Salience network model of insula function; switching between networks |
| 11 | Seeley et al. 2007 | Salience network | Dissociable salience and executive control networks |
| 27 | Dehaene, Lau & Kouider 2017 | Global workspace | Global workspace formulation of conscious access |
| 28 | Mashour, Roelfsema, Changeux & Dehaene 2020 | Global workspace | Global neuronal workspace; mechanisms of conscious processing |
| 29 | Paulus & Stewart 2014 | Interoception in addiction | Interoception and drug addiction; mechanistic framework |
| 30 | Critchley et al. 2004 | Interoception (neural systems) | Neural systems supporting interoceptive awareness |
| 31 | Naqvi, Rudrauf, Damasio & Bechara 2007 | Lesion evidence | Insular damage disrupts cigarette addiction; foundational lesion evidence |
| 32 | Joutsa et al. 2022 | Lesion-network mapping | Addiction-remission lesions map to distributed insula–ACC circuit |
| 33 | Fox 2018 | Lesion-network methodology | Lesion-network mapping methodology |
| 49 | Tononi & Koch 2015 | Integrated information theory | Integrated information theory of consciousness |
| 50 | Oizumi, Albantakis & Tononi 2014 | Integrated information theory | Integrated information theory 3.0; mechanisms of consciousness |
| 51 | Khalsa et al. 2018 | Interoception (clinical roadmap) | Interoception and mental health; research roadmap |
| 52 | Craig 2003 | Interoception (foundational) | Interoception as sense of physiological body condition |
| 53 | Seth 2013 | Interoceptive inference | Interoceptive inference, emotion, and the embodied self |
| 54 | Barrett, Quigley & Hamilton 2016 | Interoception and active inference | Active inference theory of allostasis and interoception |
| 56 | Naqvi & Bechara 2009 | Insula in addiction | Hidden island of addiction: the insula |
| 75 | Arias-García, Caballero-Cruz & García-Ortega 2023 | Integrated information theory | Systematic review of integrated information theory |
